# Tirzepatide ameliorates spatial learning and memory impairment through modulation of aberrant insulin resistance and inflammation response in diabetic rats

**DOI:** 10.3389/fphar.2023.1146960

**Published:** 2023-08-28

**Authors:** Xiying Guo, Min Lei, Jiangyan Zhao, Min Wu, Zhanhong Ren, Xiaosong Yang, Changhan Ouyang, Xiufen Liu, Chao Liu, Qingjie Chen

**Affiliations:** ^1^ Hubei Key Laboratory of Diabetes and Angiopathy, Xianning Medical College, Medical Research Institute, Hubei University of Science and Technology, Xianning, China; ^2^ Pharmacy College, Xianning Medical College, Hubei University of Science and Technology, Xianning, China

**Keywords:** tirzepatide, diabetes mellitus, spatial learning and memory, Aβ, synaptic plasticity, inflammation, insulin

## Abstract

**Background:** One of the typical symptoms of diabetes mellitus patients was memory impairment, which was followed by gradual cognitive deterioration and for which there is no efficient treatment. The anti-diabetic incretin hormones glucose-dependent insulinotropic polypeptide (GIP) and glucagon-like peptide-1 (GLP-1) were demonstrated to have highly neuroprotective benefits in animal models of AD. We wanted to find out how the GLP-1/GIP dual agonist tirzepatide affected diabetes’s impairment of spatial learning memory.

**Methods:** High fat diet and streptozotocin injection-induced diabetic rats were injected intraperitoneally with Tirzepatide (1.35 mg/kg) once a week. The protective effects were assessed using the Morris water maze test, immunofluorescence, and Western blot analysis. Golgi staining was adopted for quantified dendritic spines.

**Results:** Tirzepatide significantly improved impaired glucose tolerance, fasting blood glucose level, and insulin level in diabetic rats. Then, tirzepatide dramatically alleviated spatial learning and memory impairment, inhibited Aβ accumulation, prevented structural damage, boosted the synthesis of synaptic proteins and increased dendritic spines formation in diabetic hippocampus. Furthermore, some aberrant changes in signal molecules concerning inflammation signaling pathways were normalized after tirzepatide treatment in diabetic rats. Finally, PI3K/Akt/GSK3β signaling pathway was restored by tirzepatide.

**Conclusion:** Tirzepatide obviously exerts a protective effect against spatial learning and memory impairment, potentially through regulating abnormal insulin resistance and inflammatory responses.

## 1 Introduction

Diabetes mellitus (DM) is the most prevalent chronic metabolic disorder illness with a rising morbidity and death rate in the entire world. Many different problems are usually associated with DM. There is mounting evidence that diabetes mellitus increases the risk of dementia, cognitive impairment, or cognitive decline (Gudala et al., 2013; Baumgart et al., 2015; Magliano et al., 2019). One of the most common diabetes-related central nervous system problems is diabetic cognitive impairment, which eventually leads to Alzheimer’s disease (AD) and has a detrimental impact on patients’ families and the general public health (Shinohara and Sato, 2017). However, the exact reasons for the increased risk of cognitive impairment in people with diabetes are not fully understood. Epidemiological research demonstrates that DM and AD frequently coexist in a number of people due to comparable insulin resistance in the brain (Kim and Feldman, 2015; Stanciu et al., 2020). Additionally, the neuroprotective effects of anti-diabetic medications showed a possible connection between DM and AD (Sim et al., 2021). Sadly, there are no effective medications available right now that can stop or delay the development of cognitive loss in diabetes. In order to prevent or postpone the cognitive impairment brought on by diabetes, it is crucial to develop safe and efficient treatment medications or to investigate novel therapeutic targets.

According to mounting research, insulin resistance may impair cognitive functioning by resulting in mitochondrial malfunction, changes in synaptic plasticity, development of Aβ plaques, hyperphosphorylation of Tau, among other things (Jayaraman and Pike, 2014; Biessels and Despa, 2018). Furthermore, clinical research has shown that insulin therapy improves cognitive performance in both T2DM and AD, indicating that poor insulin signaling may be a major contributor to diabetic cognitive impairment (Novak et al., 2014; Claxton et al., 2015; Chapman et al., 2018). It is well known that hyperglycemia can trigger and promote inflammatory processes. Similarly, diabetes-related cognitive loss is also accompanied with chronic inflammatory infiltration in microglia, astrocytes, neurons, and endothelial cells (Stranahan et al., 2016; Xu et al., 2021). Moreover, inhibition of neuroinflammation can alleviate cognitive dysfunction in diabetic rats (Kuwar et al., 2021). It is indisputable that the inflammatory cytokines are produced in response to neurodegeneration (Li et al., 2016; Lee et al., 2022; Salvador et al., 2022). According to recent studies, neuroinflammation is a significant component that promotes the growth of important pathogenic proteins in the brain and contributes to the deterioration of cognitive function (Leng and Edisom, 2021; Pascoal et al., 2021). Due to this, we investigate in our experiments if tirzepatide offers a protective effect against cognitive impairment by repairing the insulin signaling system and exerting an anti-inflammatory impact in the hippocampus.

Tirzepatide, the first dual GIP and GLP-1 receptor agonist for human treatment, can significantly reduce blood glucose levels, improve insulin sensitivity, and has a remarkable effect on patients with cardiovascular and cerebrovascular diseases (Hammoud and Drucker, 2022; Sattar et al., 2022). LIn addition to significantly lowering HBA1c over time, tirzepatide therapy also promotes considerable weight reduction and enhances lipid metabolism (Azuri et al., 2023; Lingvay et al., 2023). Additionally, GLP-1 receptor agonists (RAs) have direct impacts on synaptic (Wang et al., 2013) and microglial (Spielman et al., 2017) activities as well as neuroprotective qualities by reestablishing brain energy metabolism (Daniele et al., 2015; Gejl et al., 2017). GIP receptor activation has similar protective properties as GLP-1 receptor activation, and that improving GIP signaling in the brain may be protective in AD (Ji et al., 2016). As receptors of GIP and GLP-1 are both discovered in Central Nervous System (CNS) (Gabe et al., 2020), and recent study shows that the same novel GLP-1/GIP receptor agonist DA4-JC shows neuroprotective effect in the different mouse model of AD (Maskery et al., 2020; Cai et al., 2021). However, it is currently unknown and has to be further elucidated whether tirizepatide can directly affect hippocampal neurons through the blood-brain barrier, and has the neuroprotective ability to modulate the inflammatory response and the insulin signaling pathway to prevent DM-induced memory and cognitive decline.

Therefore, the current study set out to better understand the molecular mechanisms behind tirzepatide’s neuroprotective effects on the impairments of spatial learning and memory in diabetic rats caused by STZ injection and high-fat diet. Our results showed that tirzepatide improved abilities with spatial learning and memory, possibly by lowering inflammatory reactions and, to some extent, through modifying the insulin signal pathway. These events give us a framework for understanding how tirzepatide works to cure cognitive impairment in the hippocampus. These findings demonstrated tirzepatide’s neuroprotective properties against the pathophysiology of cognitive impairment brought on by DM, and they also raised the possibility that GIP/GLP-1 receptors would be a useful therapeutic target for avoiding the central nervous system difficulties brought on by DM.

## 2 Materials and methods

### 2.1 Animals

**FIGURE 1:**
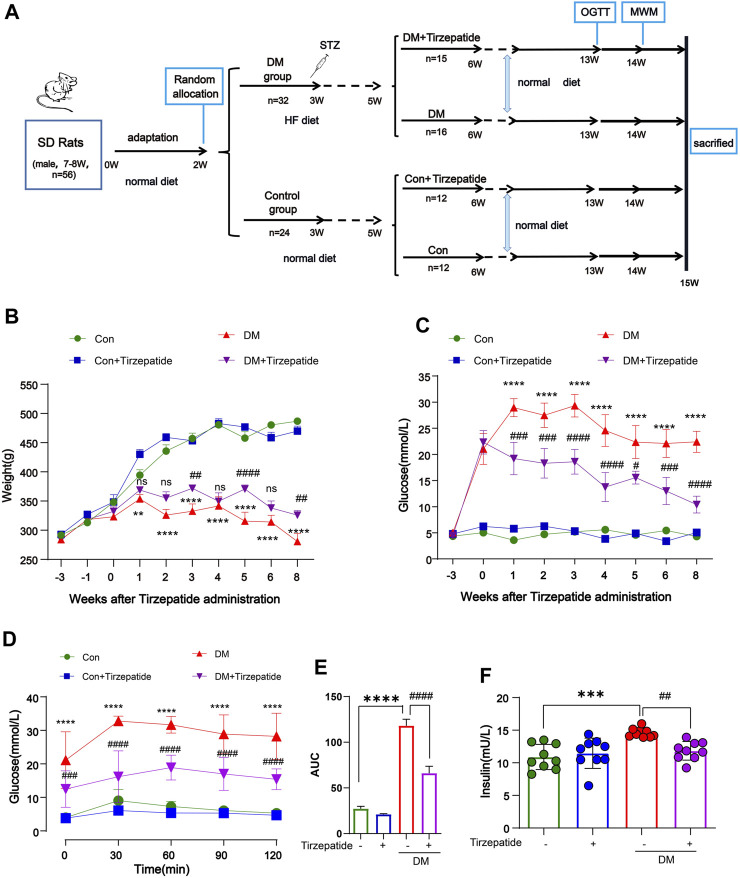
Tirzepatide ameliorated hyperglycemia and insulin resistance in diabetic rats. **(A)** Flow chart of animal experiment design. **(B)** Body weight and **(C)** Fasting plasma glucose levels variation, n = 8/group. **(D)** The glucose tolerance test at the 13th week, n = 8/group. **(E)** The AUC of blood glucose curves in OGTT. **(F)** The plasma insulin levels were measured using ELISA kits, n = 9/group. The data represent the mean ± SD. ^**^ *p* < 0.01 DM vs Con; ^***^ *p* < 0.001 DM vs Con; ^****^ *p* < 0.0001 DM vs Con; ^*##*^ *p* < 0.01 DM + Tirzepatide vs DM; ^*###*^ *p* < 0.001 DM + Tirzepatide vs DM; ^*####*^ *p* < 0.0001 DM + Tirzepatide vs DM.

Male Sprague Dawley rats weighing between 180 and 200 g (aged 7–8 weeks) were obtained from Beijing Vital River Laboratory Animal Technology Co., Ltd. (China) [Certificate: SCXK (Beijing) 2016-0011]. Rats were raised in Specific Pathogen Free (SPF) conditions with a light/dark cycle of 12 h/12 h and temperature–humidity (22°C ± 1°C, 50% ± 10%) controlled. All procedures were approved by the Animal Care and Use Committee of Hubei University of Science and Technology, Xianning, China (IACUC Number: 2021-03-003). Animal care and handling were performed according to the Declaration of management of laboratory animals regarding the care and use of laboratory animals. After 2 weeks adaptation with normal diet, a total of 32 rats were fed with HF diet (67.5% standard laboratory rat chow, 20% sugar, 10% lard, 2% cholesterol and 0.5% bile salts), while 24 rats were raised by standard chow. According to our previous study, 35 mg/kg STZ was injected by intraperitoneal injection in the rats of HF diet group, whereas normal group were injected with citrate buffer only. After 2 weeks feeding, 31 rats with a fasting blood glucose levels reaching 11.0 mmol/L were randomly divided into two experimental groups as follows: diabetes mellitus group (DM), DM + Tirzepatide group (Tirzepatide, 1.35 mg/kg, once a week). At the same time, 24 rats of standard chow group were randomly divided into control group (Con) and Con + Tirzepatide group (Tirzepatide, 1.35 mg/kg, once a week). Tirzepatide (LY3298176) was obtained from Merck (United States). All drugs were prepared preserving more than 1 year under given conditions avoiding degradation. Oral glucose tolerance test (OGTT) was performed on the 13th week. Behavioral test was conducted before the sacrificed week. Fasting blood glucose and body weight were measured weekly until the sacrificed week. In the 15th week, all rats were sacrificed and collected samples which were executed follow-up experiments. A timeline of experimental procedure is presented in Figure 1A.

### 2.2 Oral glucose tolerance test (OGTT)

After fasting for 12 h (20:00–8:00), the rats were administered a solution of glucose (2.0 g/kg body weight) by oral gavage. The blood glucose levels in the tail were obtained before (0 min) and 30 min, 60 min, and 120 min after glucose gavage by a commercial Blood Glucometer (Sinocare, China).

### 2.3 Morris water maze (MWM)

The Morris water maze test is extensively used to assess spatial learning and memory status of the rodents. Behavioral testing started before sacrificed and 10 rats of each group were observed. We adapted the protocol described in Wang et al., 2019. The Morris water maze is a white circular pool with video capture system (150 cm diameter × 70 cm depth, temperature 22°C ± 1°C) which was segmented into four quadrants at surface. A white escape platform was placed in the middle of the second quadrants at the depth of 1–2 cm below the water level. On days 1–5, the rats were conducted for the 5-days consecutive training trials. During the training period, the rats were randomly placed into the first quadrants as start locations to find the hidden platform within 90s. If failed, the rats were guided to locate the platform and stayed for 15s. At the 6th day, the platform was removed to conduct the memory retention test, and the rats were also randomly placed into the first quadrants and freely swam for 90s (probe trial). All the swimming trials were automatically monitored with a camera above 2 m from water level. Monitoring files were subsequently analyzed to obtain the latency to locate the platform, the time spent in each quadrant, the number of rats crossing the platform position and the swimming speed. MWM assay and data analysis were conducted by an unwitting observer.

### 2.4 Protein extraction and western blot

The hippocampus were quickly dissected and homogenized by ice-cold RIPA buffer (50 mM Tris-HCl, pH 7.5; 150 mM NaCl; 1 mM EDTA; 1% NP-40; 0.1% SDS; and 1% Triton X-100) contained with 0.1% phosphatase inhibitor and 0.1% protease inhibitor cocktail. The homogenates were centrifuged at 4°C and then the supernatants were boiled with 1 × loading buffer for 5 min at 100°C. Protein extractions were quantified by BCA Protein Assay Kits in advance. A total of 20 μg of protein extractions were separated on 10% SDS-polyacrylamide gel electrophoresis and then transferred onto a 0.45 μm PVDF membrane. After blocking in TBST buffer (10 mM Tris-HCl, pH 7.5; 150 mM NaCl; 0.1% Tween 20) within 5% skimmed milk for 1 h at room temperature, the membrane was incubated with specific primary antibodies overnight at 4°C and then washed three times for 10 min with TBST. Finally, a corresponding horseradish peroxidase-conjugated secondary antibody was utilized to incubate the membrane for 2 h at room temperature. Specific signals expected were detected by Bio-Rad Exposure System through ECL. The results were measured by normalizing the intensities of target bands to their corresponding housekeeping genes bands with Fiji analysis software. The dilution ratio and manufacturer of antibodies which used are shown in Table 1. Polyvinylidenedifluoride (PVDF) membrane (0.45 µm) were obtained from Millipore (United States). All other reagents purchased from located market were analytical grade.

**TABLE 1:** Antibodies for western blot.

Name	Manufacturer	Catalog number	Dilution ratio
IKKα	Affinity	AF6014	1/1000
p-IKKα	ABclonal	AP0505	1/1000
PSD95	ABclonal	A7889	1/1000
SYTl	ABclonal	AO992	1/2000
PI3K	Proteintech	60225-1-lg	1/3000
p-PI3K	Affinity	AF3242	1/1000
AKT	Proteintech	60203-2-lg	1/3000
p-AKT	ABclonal	AP1266	1/1000
GSK3β	ABclonal	A2081	1/1000
p-GSK3β	ABclonal	AP0039	1/1000
IR	ABclonal	A19067	1/3000
p-IRSl	Affinity	AF3272	1/1000
APP	ABclonal	A162655	1/1000
BACEl	ABclonal	A5266	1/1000
GAPDH	Servicebio	GB11002	1/2000
Tubulin	ABclonal	A12289	1/2000
HRP Goat Anti-Rabbit IgG	Servicebio	BL003A	1/10000
HRP Goat Anti-Mouse IgG	Proteintech	SA0000l −1	1/10000

### 2.5 RNA isolation and real-time polymerase chain reaction (RT-PCR)

Total RNA was isolated from 30 mg dissected hippocampal tissues with TriZol reagent (Servicebio, China) and chloroform. The reverse transcription of total RNA into cDNA was finished with a Super Script III reverse transcriptase Kit (Servicebio, China). The resulting cDNA was 10 times diluted with DNase-free water and subsequently quantified by RT-PCR with a Fast Start universal SYBR Green Master (ROX) PCR kit (Servicebio, China). All data expressing was calculated the relative ratio of the target gene to the housekeeping gene *β*-actin. The following primers have been used for RT-PCR:

TNF-α: Forward-CCCCAGGGACCTCTCTCTAA, Reverse-TGAGGTACAGGCCCTCTGAT;

IL-6: Forward-ACAGGGAGAGGGAGCGATAA, Reverse-GAGAAGGCAACTGGACCGAA;

IL-1β: Forward-CGATGCACCTGTACGATCAC, Reverse-TCTTTCAACACGCAGGACAG;

β-actin: Forward-GGACTCCTATGTGGGTGACGAG, Reverse-TCACGGTTGGCCTTAGGGTT.

### 2.6 Nissl’s staining

Nissl’s staining was performed by Servicebio. Images were obtained under a fluorescence microscope (Olympus) with a 10 × objective lens and a 40 × objective lens.

### 2.7 Immunofluorescence

Brain slices at the same level and region were randomly selected and conducted with the following procedures: first, membranes were dissolved with 0.5% Triton-X for 10 min. After blocking non-specific protein binding sites with 5% bovine serum albumin (BSA) for 1 h at room temperature, the slices were then incubated with specific primary antibodies diluted overnight at 4°C. Subsequently, after rinsing three times for 5 min with PBS, the brain slices were incubated in the dark with the fluorochrome-conjugated secondary antibodies for 2 h at room temperature. Finally, the brain slices were incubated with DAPI for 5 min at room temperature. Observation was completed under a inverted fluorescence microscope (Olympus) with a 40 × objective lens. The dilution ratio and manufacturer of antibodies which used are shown in Table 2.

**TABLE 2:** Antibodies for immunofluorescence.

Name	Manufacturer	Catalog number	Dilution ratio
p-NF-KB	Cell Signaling Technology	Q04206	1/500
Aβ40	Servicebio	P12023	1/500
NeuN	Proteintech	66836-1-Ig	1/300
FITC Goat Anti-Mouse IgG	Servicebio	AS00l	1/300
Cy3 Goat Anti-Rabbit IgG	Servicebio	AS007	1/300

### 2.8 Golgi staining and dendritic spine analysis

Brain tissues at the same region were quickly taken into the Golgi solution (2.5% K2Cr2O7; 2.5% HgCl2; 2% K2CrO4) and stored in the dark for 14 days, the fresh Golgi solution was changed every 2 days. Then, tissues were replaced into 30% sucrose buffer at 4°C until sunk. Slices (200 μm) were obtained by scillating microtome (LC 2000, Leica) and conducted with the following procedures: first, the slices were washed by distilled water for 1 min and soaked in NH4OH for 30 min in the dark. After fixed with Kodak fixative for 30 min in the dark, the slices were then dehydrated with ethanol with different concentration gradients (50% ethanol for 1 min; 70% ethanol for 1 min; 95% ethanol for 1 min; 100% ethanol for 5 min, 3 times). Subsequently, the brain slices were incubated in CXA solution (chloroform: xylene: ethanol = 1:1:1 Vol) for 15 min. Finally, neutral balsam mounting medium was used to mount the slices. Laser confocal microscope (OLYMPUS FV3000, Japan) with a 10 × objective lens, a 20 × objective lens and a 40 × objective lens was employed for image collection. The images were analyzed and processed by Fiji analysis software. For each selected dendritic branch, the length measurement was at least 10 μm. The dendritic spine density was counted as the number of spines per 10 μm at the selected dendritic branch (Cheng et al., 2022).

### 2.9 Statistical analysis

Data were analyzed by GraphPad Prism 8 software and expressed as mean ± SD. Statistical significance was evaluated by Two-way ANOVA. *p* values <0.05 indicated that significant level was existed.

## 3 Results

### 3.1 Tirzepatide ameliorated hyperglycemia and insulin resistance in diabetic rats

One week after the STZ injection, the blood glucose assessment identified total 31 out of the 32 HF diet-fed rats developed diabetes for the further study. As expected, from the forth week after STZ injection, the body weight of diabetic rats no longer changed significantly, but the control group continued to grow (Figure 1B). Also, STZ injection increased the fasting blood glucose level from ∼5 mM to ∼22 mM within 2 weeks (Figure 1C). These results validated the success of diabetic rats modeling by HF diet and STZ injection. Compared with the DM group, tirzepatide treatment produced a significant reduction in fasting blood glucose (Figure 1C) and plasma insulin (Figure 1E), but a little increase in body weight (Figure 1B), indicating the beneficial effects of tirzepatide on glucose metabolism as expected.

We further adopted OGTT to evaluate hypoglycemic effects of tirzepatide (Figure 1D). The blood glucose curve of the DM + Tirzepatide group raised in 0–60 min and declined then, whereas other three groups raised in 0–30 min and declined then. Moreover, curves of DM + Tirzepatide group were below the DM group. Further, the AUC of the curves was significantly increased in DM rats but reduced after tirzepatide treatment (Figure 1E), indicating that tirzepatide improves glucose homeostasis in diabetic rats. These results may exhibit the tirzepatide potential ability of glycemic control without hypoglycaemia (Lingvay et al., 2023).

After sacrificed, we collected serum to detect insulin levels (Figure 1F). The serum insulin level was strikingly increased in DM rats but normalized after tirzepatide treatment, suggesting tirzepatide conduced to the amelioration of insulin resistance in diabetic rats.

### 3.2 Tirzepatide ameliorated DM-induced spatial learning and memory impairment

**FIGURE 2:**
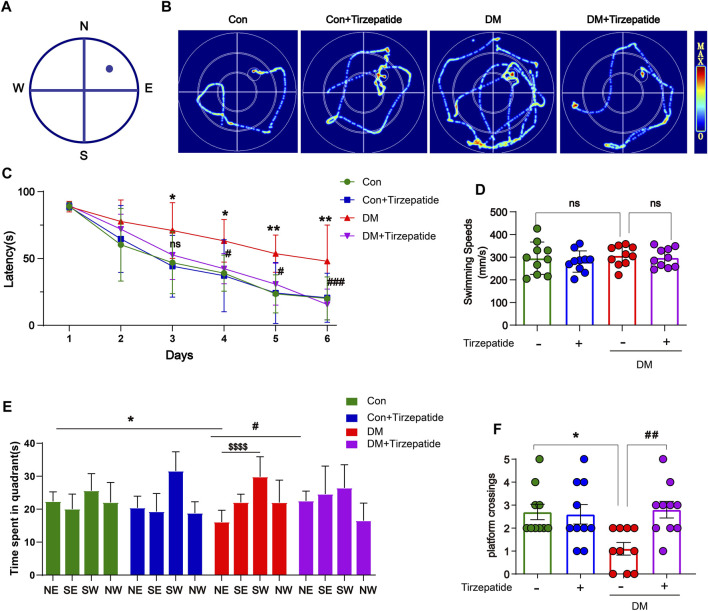
Tirzepatide ameliorated DM-induced Spatial learning memory impairment. **(A)** MWM schematic. **(B)** The trajectory of rats in MWM. **(C)** The time spent to find the platform (latency) in the 5-days consecutive training trials and the memory retention trial. **(D)** The swimming speed analysis **(E)** the time spent in four quadrants and **(F)** the platform crossing during the memory retention trial. n = 10/group. The data represent the mean ± SD. ^*^ *p* < 0.05 DM vs Con; ^***^ *p* < 0.001 DM vs Con; ^*#*^ *p < 0.05* DM + Tirzepatide vs DM; ^*##*^ *p* < 0.01 DM + Tirzepatide vs DM; ^*$$$$*^ *p* < 0.0001 NE vs SW.

To ascertain whether tirzepatide may improve the learning and memory impairment associated with the hippocampus in diabetic rats, we used the MWM test, which involves training rats with a concealed platform to gauge their capacity for spatial learning and memory (Luchsinger, 2012) (Figures 2A, B). Results showed that the DM + Tirzepatide group rats performed better than DM animals in learning the position of the escape platform during the training trails and the memory retention experiments (Figure 2C). The escape latency in DM rats was longer than Con rats, but was effectively reduced after tirzepatide treatment, when the average swimming speeds did not significantly changed (Figure 2D). Additionally, in the memory retention experiment, tirzepatide significantly increased the time spent in the target area and the number of platform crossings (Figures 2E, F), which were remarkably reduced in diabetic rats. These results suggest that tirzepatide may ameliorate spatial learning and memory impairment in diabetic rats.

### 3.3 Tirzepatide inhibited Aβ formation in the hippocampus of diabetic rats

**FIGURE 3:**
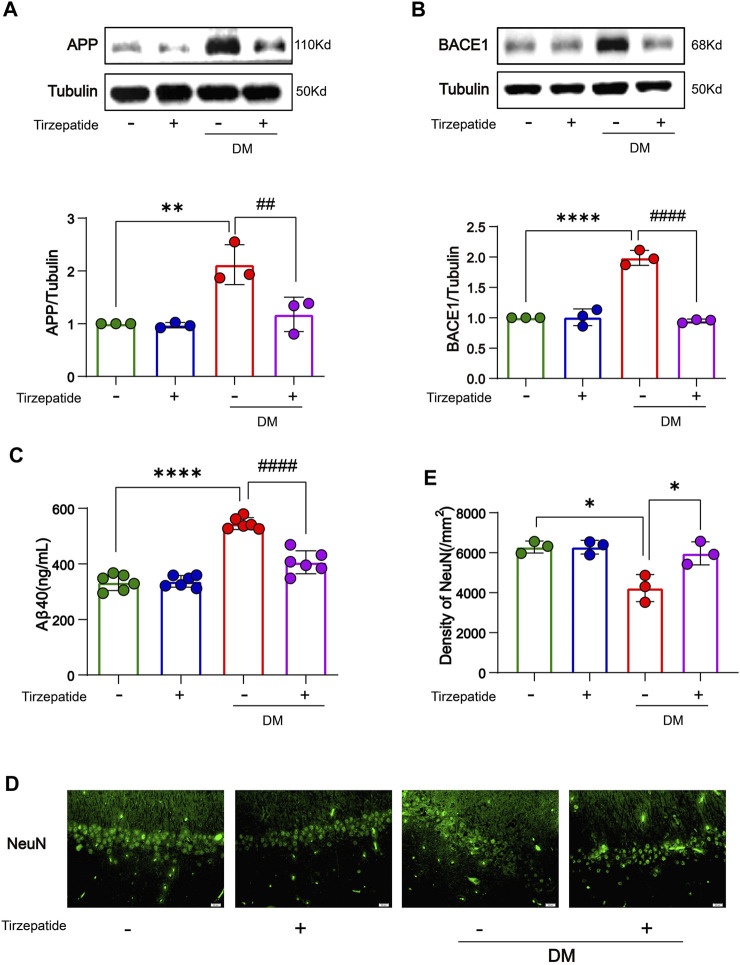
Tirzepatide improved APP misprocessing in the hippocampus. **(A)** APP and **(B)** BACE-1 expressions were assessed by Western-blot, n = 3/group. **(C)** Aβ40 levels were detected by ELISA, n = 6. **(D)** Representative immunofluorescence staining images of the CA1 neuron (NeuN) in the hippocampus. Scale bar, 20 μm. **(E)** Bar plots showing the neuron density in the hippocampus, n = 3/group. The data represent the mean ± SD. ^*^ *p* < 0.05 DM vs Con; ^**^ *p* < 0.01 DM vs Con; ^****^ *p* < 0.0001 DM vs Con; ^*#*^ *p* < 0.05 DM + Tirzepatide vs DM; ^*##*^ *p* < 0.01 DM + Tirzepatide vs DM; ^*####*^ *p* < 0.0001 DM + Tirzepatide vs DM.

Studies showed that increased glucose concentrations in brain tissue may lead to abnormal glucose metabolism in the brain, eventually leads to deposits of amyloid beta (Aβ) protein and the development of plaques which may exacerbate dementia-related neuropathology (Alafuzoff et al., 2009; Kirvalidze et al., 2022; van Arendonk et al., 2022). *β*-site amyloid precursor protein (APP) cleaving enzyme 1 (BACE1) is a rate-limiting enzyme for Aβ production (Sinha et al., 1999) and has been extensively researched for its neuronal functions (Hampel et al., 2021). We verified that the accumulated APP/BACE-1 in the hippocampus of diabetic rats (Chen et al., 2017) was significantly inhibited by tirzepatide (Figures 3A, B). Next, to investigate the effects of Tirzepatide treatment on Aβ production, Aβ40 levels were detected by an ELISA kit. The increased expression of Aβ40 in the DM group was significantly reduced after tirzepatide treatment (Figure 3C). Subsequently, the immunofluorescence of NeuN at CA1 region (Figure 3D) and Nissl’s staining at CA1, CA3, DG and hilus region (Supplementary Figure S1) were applied to show the changes in hippocampus and to determine whether there were any organic changes in the diabetic brains. Compared with the DM rats, the density of the neurons was greatly improved by Tirzepatide (Figure 3E). These results suggest that tirzepatide treatment can effectively inhibit Aβ formation and neurons loss in the hippocampus of diabetic rats.

### 3.4 Tirzepatide affected synaptophysin proteins and dendritic spines in diabetic rats

**FIGURE 4:**
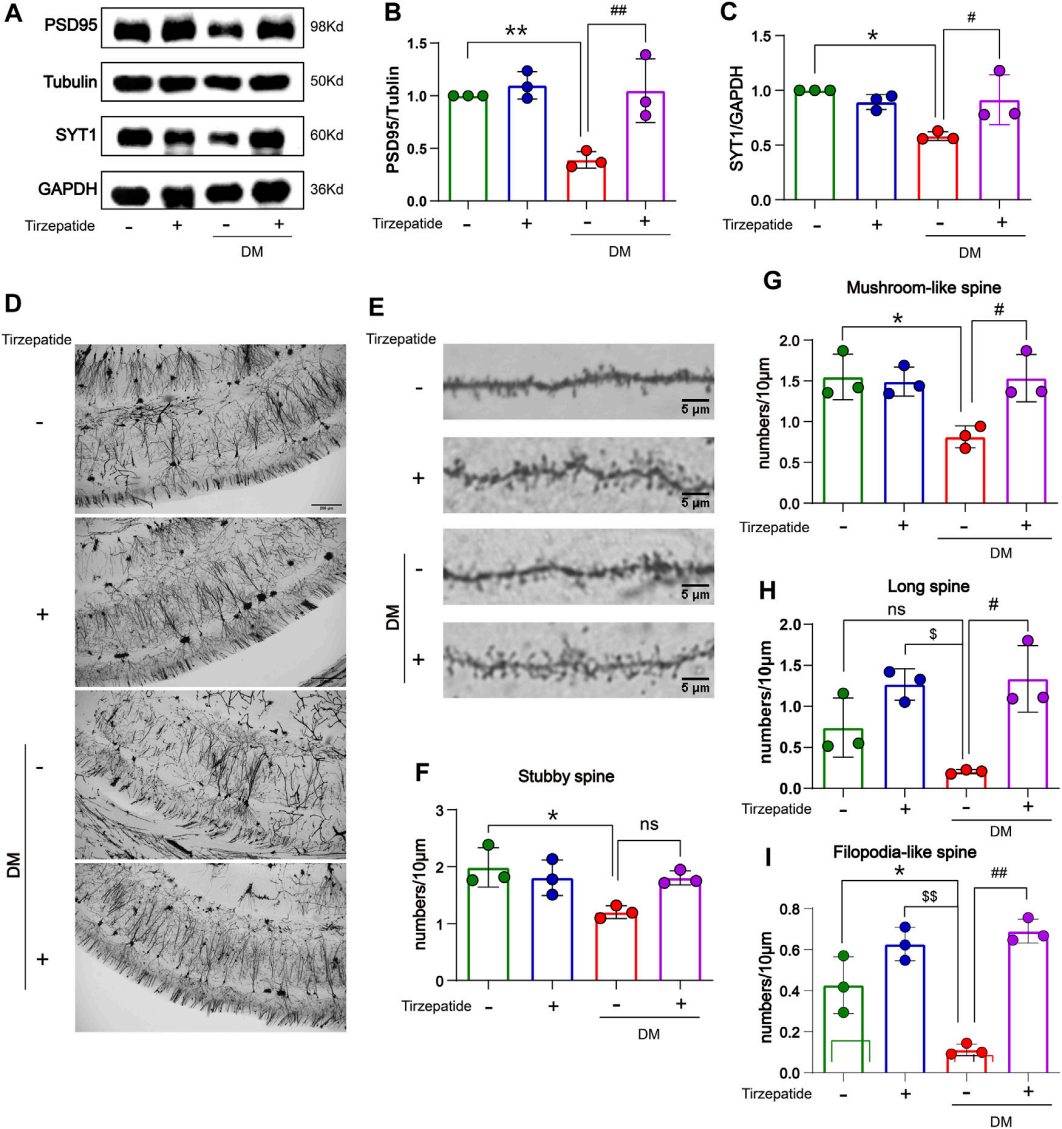
Tirzepatide affected synaptophysin proteins and dendritic spines in diabetic rats. **(A–C)** PSD95 and SYT1 levels were detected by Western blot, n = 3/group. **(D)** Representative images of neurons in the CA1 region; scale bar, 200 μm. **(E)** Representative images of dendritic spines; scale bar, 5 μm. Statistics of **(F)** Stubby spine, **(G)** Mushroom-like spine **(H)** Long spine and **(I)** Filopodia-like spine, n = 3/group. The data represent the mean ± SD. ^*^ *p* < 0.05 DM vs Con; ^**^ *p* < 0.01 DM vs Con; ^***^ *p* < 0.001 DM vs Con; ^****^ *p* < 0.0001 DM vs Con; ^*$*^ *p* < 0.05 con + Tirzepatide vs DM; ^*$$*^ *p* < 0.01 con + Tirzepatide vs DM; ^*#*^ *p* < 0.05 DM + Tirzepatide vs DM; ^*##*^ *p* < 0.01 DM + Tirzepatide vs DM; ^*####*^ *p* < 0.0001 DM + Tirzepatide vs DM.

Hippocampal synaptophysin proteins were significantly responsible for the ability of spatial learning and memory, whereas memory loss was associated with a decline in synaptophysin protein levels (Yao et al., 2004; Xu et al., 2019). The postsynaptic density protein 95 (PSD95) is one of the major scaffold proteins of the dendritic spines and determines the synaptic response (Okabe, 2007). Synaptotagmin-1 (SYT1) is a synaptic vesicle protein that is responsible for rapid release in hippocampal synapses through its calcium sensing activity (Geppert et al., 1994; Bowers and Reist, 2020). Therefore, we tested the expression of PSD95 and SYT1 in the hippocampus. In our results, PSD95 and SYT1 were decreased in DM rats, but rescued after tirzepatide treatment (Figures 4A–C), suggesting tirzepatide may possess ability to affect on synaptic function via synaptophysin proteins. Dendritic branches in the CA1 region of hippocampus and dendritic spines were observed under high magnifications of the dendritic arbor (Figures 4D, E). Dendritic spines form synapses and participate in the transmission of neurotransmitters, and are thought to be substrates for motor learning and memory (Hering and Sheng, 2001). Then we counted the four different types of dendritic spines: stubby-spine, mushroom-spine, long-spine, and filopodia-like spine. After tirzepatide treatment, mushroom-spine, long-spine, and filopodia-like spine expect stubby-spine exhibited considerably more than DM group (Figures 4F–I), implying that there might be a potentiation in synaptic function. These results suggested that tirzepatide may play a role in synaptic protein synthesis and dendritic spines formation through potential mechanisms, thus improving the spatial learning and memory ability of diabetic rats.

### 3.5 Tirzepatide inhibited hippocampal inflammatory activity in diabetic rats

**FIGURE 5:**
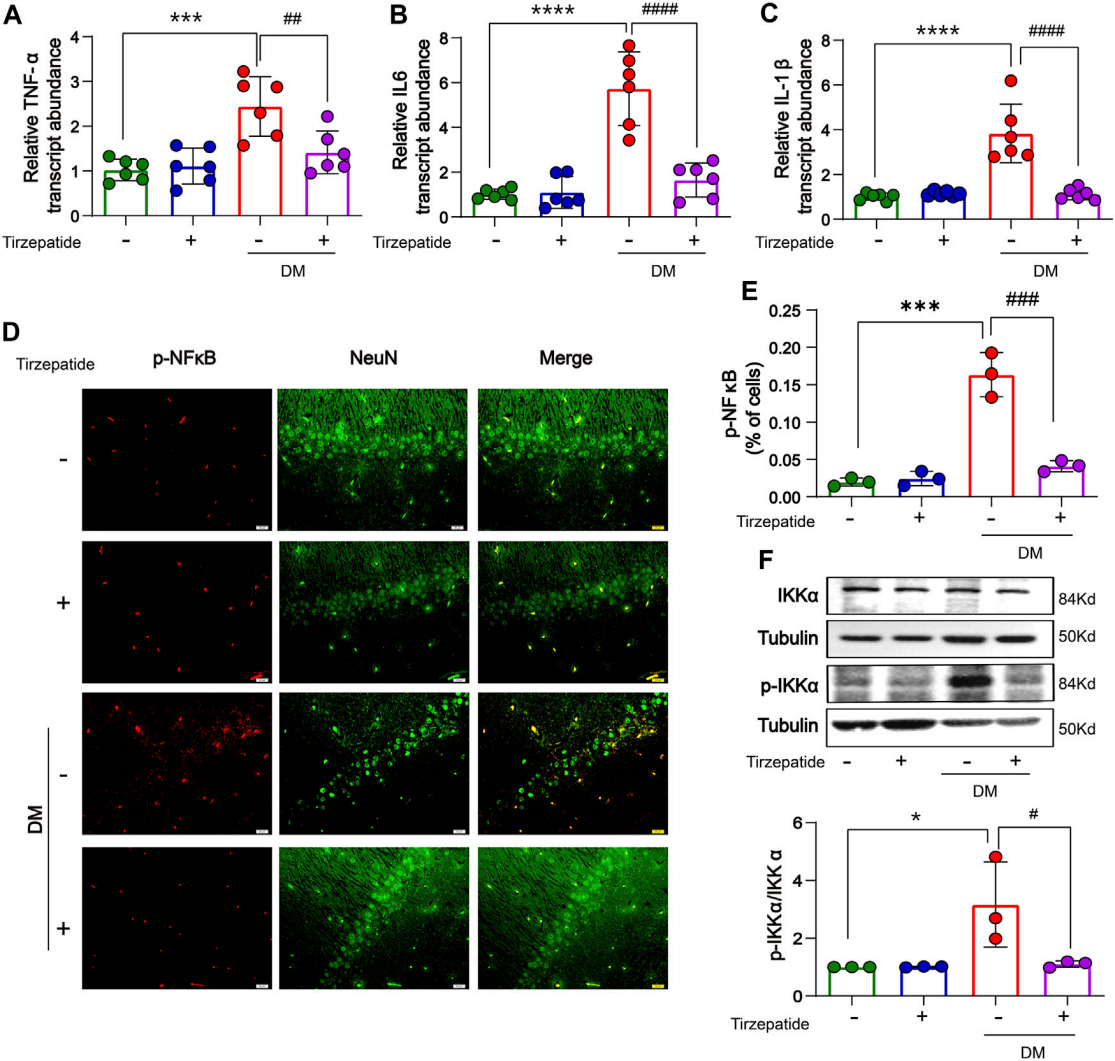
Tirzepatide inhibited inflammatory response in diabetic rats. The mRNA expression of **(A)** TNF-α, **(B)** IL-6 and **(C)** IL-1β were reduced by tirzepatide, n = 6/group. **(D)** Representative immunofluorescence images showing p-NFκB in the CA1 region of hippocampus. Scale bar, 20 μm. **(E)** The % of cells with p-NFκB immunoreactivity was inhibited after tirzepatide, n = 3/group. **(F)** Tirzepatide prevented the phosphorylation of IKKα, n = 3/group. The data represent the mean ± SD. ^*^ *p* < 0.05 DM vs Con; ^***^ *p* < 0.001 DM vs Con; ^****^ *p* < 0.0001 DM vs Con; ^*#*^ *p* < 0.05 DM + Tirzepatide vs DM; ^*##*^ *p* < 0.01 DM + Tirzepatide vs DM; ^###^ *p* < 0.001 DM + Tirzepatide vs DM; ^*####*^ *p* < 0.0001 DM + Tirzepatide vs DM.

Systemic or local inflammation is caused by abnormal glucose transport and metabolism in the peripheral or CNS, which aids in the development of DM (Jorge et al., 2011; Muriach et al., 2014). The memory ability in diabetic rats is impaired by the inflammatory response in the middle prefrontal cortex (Wang et al., 2019). To determine whether tirzepatide have anti-inflammation effects in hippocampus, RT-PCR was selected to appraise the mRNA levels of TNF-α, IL-6 and IL-1β. Results showed an increment was existed in diabetic rats when compared with Con group, whereas significantly inhibited by tirzepatide (Figures 5A–C). Based on these observations, we adopted the immunofluorescence assay to investigate the expression levels of phosphorylated NF-κB. Similarly, the levels were augmented in DM group, and reduced after tirzepatide treatment (Figures 5D, E). Next we observed that the phosphorylation levels of IKK were greatly increased in DM group when compared with Con. Similarly, the augment was strongly suppressed after tirzepatide adminstration (Figure 5F). These results indicated that tirzepatide receded the generation of inflammation in the hippocampus of diabetic rats.

### 3.6 Tirzepatide reduced the insulin resistance and regulated related proteins in the insulin signaling pathway in hippocampus of diabetic rats

**FIGURE 6:**
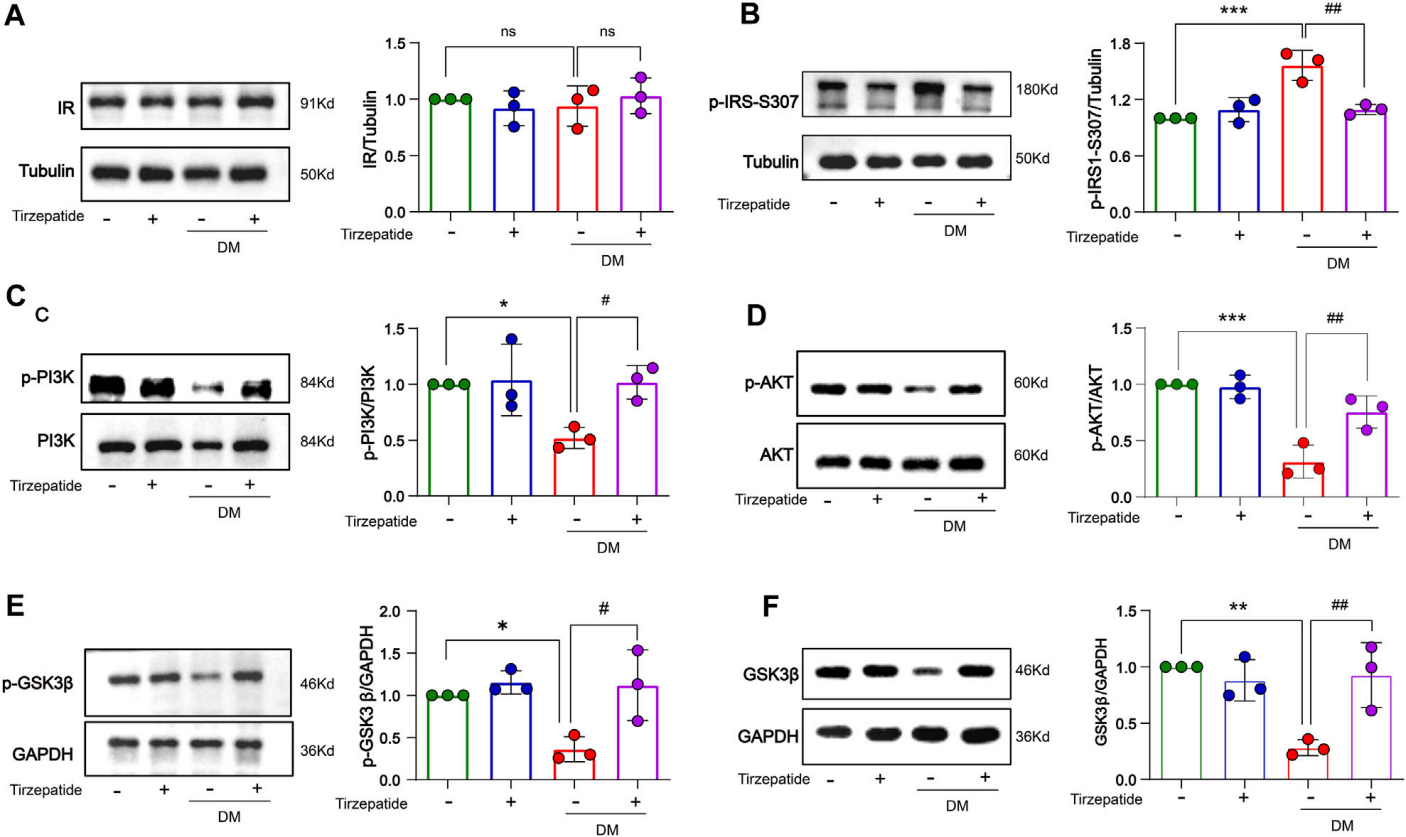
Tirzepatide improved insulin signaling pathway in diabetic rats. **(A)**The protein expression levels of IR were similar in four groups, n = 3/group. The phosphorylation of **(B)** IRS-1 ser307, **(C)** PI3K **(D)** AKT, **(E)** GSK3β were improved in diabetic rats after tirzepatide treatment, n = 3/group. **(F)** The protein expression levels of GSK3β in hippocampus were increased by tirzepatide in diabetic rats, n = 3/group. The data represent the mean ± SD. ^*^ *p* < 0.05 vs Con; ^**^ *p* < 0.01 DM vs Con; ^***^ *p* < 0.001 DM vs Con; ^*#*^ *p* < 0.05 DM + Tirzepatide vs DM; ^*##*^ *p* < 0.01 DM + Tirzepatide vs DM.

The enhanced inflammatory activity can trigger insulin resistance in DM (Wang et al., 2018). We detected related proteins to analyze the changes of insulin signaling pathways in hippocampus. There were no remarkable changes on insulin receptor (IR) protein levels in all of four groups (Figure 6A). However, the expression of p-IRS-Ser307 was remarkably increased in DM group, while tirzepatide significantly reduced the increment (Figure 6B). Next, we detected the activation of PI3K/AKT/GSK3β signaling pathway. These results revealed that the phosphorylation levels of PI3K, AKT and GSK3β were significantly reduced in DM rats, but were normalized by tirzepatide (Figures 6C–E). Similarly, DM group remarkably reduced PI3K and GSK3β expression when compared with Con group, however tirzepatide obviously inhibited the downregulation (Figure 6F), suggesting that tirzepatide may ameliorate insulin resistance in the hippocampus of diabetic rats.

## 4 Discussion

According to the International Diabetes Federation (IDF), type 2 diabetes mellitus (T2DM) is a kind of non-communicable disease with 537 million people affected worldwide in 2021 (Centre Européen D’Étude Du Diabète Les Chiffres du Diabète., 2022). IDF and World Health Organization (WHO) predict this figure to increase to 643 million people with diabetes by 2030 and 784 million by 2045 (International Diabetes Federation IDF Diabetes Atlas|Tenth Edition., 2022). DM has become a big challenge to both financial and health. Accumulating epidemiological evidences suggested that diabetes mellitus (DM) patients were at an increasing risk of developing cognitive dysfunction (Petrova et al., 2010; Baumgart et al., 2015). Consistently, experimental researches demonstrated cognitive decline in STZ-induced diabetic rats (Bogush et al., 2017). Some neuronal pathological changes occurred in neurodegeneration disease with cognitive decline, including synapse plasticity deprivation (Duncan and Valenzuela, 2017), neuronal loss (Spangenberg et al., 2016), and decrements in dendritic spine and arborization density (Maiti et al., 2021). Abnormal gene expression in some brain areas may also alter the learning and memory ability (Konopka, 2011; Brault et al., 2021).

Due to a number of their defining properties, the incretins GLP-1 and GIP represent intriguing therapeutic possibilities for the treatment of neurodegenerative disorders. Most incretin mimics pass the blood–brain barrier with ease (Kastin et al., 2003; Hunter et al., 2012). Moreover, a number of GLP-1 mimics are already on the market to treat T2DM, and when used regularly, they show few negative effects (Campbell et al., 2013). Tirzepatide, a dual GIP and GLP-1 receptor agonist, has a variety of uses, including improving insulin sensitivity, lowering blood sugar levels, and preventing cardiovascular and cerebrovascular illnesses. (Hammoud and Drucker, 2022; Sattar et al., 2022). However, the molecular regulation process and the efficacy of the therapy still need further investigation. Here, we discovered that DM-induced inflammation, hyperglycemia, and insulin dysregulation may disrupt synaptic function and impair learning and memory. Tirzepatide, on the other hand, primarily enhanced synaptic function by reducing insulin resistance and inflammation to address spatial learning and memory impairment.

*In vitro* models of diabetes-related AD, glucagon-like peptide-1, which was created to treat Type 2 DM, greatly increased neuroprotection against advanced glycation end product-induced neuronal insult (Biswas et al., 2008; Abd El-Rady et al., 2021). The MWM assay proved that tirzepatide had the amelioration of the cognitive function (Figure 2). And the imaging of Nissl’s staining (Supplementary Figure S1) and NeuN’s immunofluorescence (Figure 3D) revealed that tirzepatide protected the neuronal damage in diabetic hippocampus’ CA1, CA3, DG, and hilus areas, which is also consistent with the role of liraglutide (Hölscher C, 2014). It is believed that the buildup of Aβ contributes to the beginning of neurodegeneration and cognitive impairment (Guo et al., 2021). BACE1, a crucial enzyme that extracts the plaque-forming Aβ peptides from the APP, has long been regarded as a standard AD target (McDade et al., 2021). In DM, altered BACE1 expressions and/or activity were frequently found (Bao et al., 2021). In our work, we noticed that the diabetic hippocampus showed signs of a buildup and an elevation in APP/BACE1 (Figures 3A, B). These results clearly showed that diabetic rats’ spatial learning and memory were impaired. Thankfully, tirzepatide effectively reduces the disability, and later reductions in Aβ40 synthesis and neuronal loss by tirzepatide supported this (Figures 3C–E).

Synaptic function affects how cognitive disorders are pathogenized (Balietti et al., 2012). PSD95 and SYT1 are significant synaptic strength and function parameters (Masliah et al., 2001; Yao et al., 2004; Xu et al., 2019). In this study, we demonstrated that tirzepatide reversed PSD95 and SYT1 loss in the hippocampus of diabetics (Figures 4A–C), suggest that tirzepatide’s ability to restore memory is related to its effect on synaptic function. Additionally, the quantity and form of dendritic spines as well as variations in dendritic branches are largely responsible for controlling synaptic function. Higher numbers of dendritic branches are frequently means memory ability improvement (Liu et al., 2008; Orellana et al., 2018), and cognitive decline is usually accompanied by dendritic branches reduction (Mehder et al., 2020; 2021; Mendell et al., 2020). In the present studies, the Golgi staining used in the current investigations revealed that the DM rats had fewer dendritic branches and spines than the Con group (Figures 4D, E), suggesting a deterioration in cognitive performance. Small spines (filopodial like-spines and long-spines) and large spines (stubby-spines and mushroom like-spines), two subtypes of dendritic spines, were categorized (Parnass et al., 2000; Grutzendler et al., 2002; Trachtenberg et al., 2002). Small spines have a possibility to grow into large spines during the learning process (Kasai et al., 2003). Large spines’ shorter and wider necks make it easier for information to go between the spines and dendrites more quickly (Tonnesen et al., 2014). The structural basis for improved learning and memory was the increased density of large dendritic spines (Cheng et al., 2022). According to our research, tirzepatide promotes the growth of both large and small dendritic spines (Figures 4F–I), which suggests improved learning and memory.

Neurodegeneration is partly contributed to the environment which is affected by cascading processes collectively termed neuroinflammation during disease (Ransohoff, 2016). Therefore, suppression of neuroinflammation would theoretically slow the development of neurodegenerative disease (Bao et al., 2019). Research showed that diabetes-associated cognitive decline had been limited by suppressed inflammation in rats (Tian et al., 2016). We wondered whether tirzepatide improves cognitive impairment through anti-inflammatory effects (Figure 5). Our results exhibit that mRNA levels of IL-1β, IL-6 and TNF-α were notably increased in the hippocampus of diabetic rats. The downstream molecules of inflammatory signaling pathways, the phosphorylation of NF-κB and IKKα, were also upregulated in hippocampus. Tirzepatide reduced the L-1β, IL-6, TNF-α and p-NFκB levels, and IKKα phosphorylation, implying that this drug may attenuate inflammation in the hippocampus of DM rats.

Inflammation usually tampered the insulin signaling pathway, and aberrant insulin signaling pathway usually exacerbated the inflammation production in turn (Nandipati et al., 2017; Petersen and Shulman, 2018). Long-term insulin resistance in the brain causes the development of AD and Aβ plaque, and which also occurs in DM (Kim et al., 2015). Our results revealed that no distinctive changes of IR in the four groups (Figure 6A). However, phospho-Ser307 in IRS-1 was increased in DM group which was inhibited by tirzepatide (Figure 6B). Serine hyperphosphorylation of IRS-1 can reduce its ability to attract PI3-kinase, resulting in blocked insulin signaling interruption (Draznin, 2006). As our expected, inhibited PI3K/AKT/GSK3β signaling pathway of diabetic-induced was remarkably activated by tirzepatide (Figures 6C–F). In previous studies, the cognitive impairment in diabetic rats was improved through preventing the generation of Aβ and stimulating the PI3K/Akt signaling pathway (Chen et al., 2017; Wang et al., 2019). Collectively, our findings demonstrated that tirzepatide has the potential of alleviating the insulin signaling deficits in diabetic hippocampal tissue.

In conclusion, our study uncovers that tirzepatide may primarily improve the aberrant inflammation activities and regulate part in ordinate proteins involved in insulin signaling pathway, thereby facilitating numerous dendritic spines production and elevating synaptic plasticity, and finally ameliorate spatial learning and memory impairment. Extensive experiments need to be executed in future to determine the precise molecular mechanisms underlying the effect of tirzepatide.

## Data Availability

The original contributions presented in the study are included in the article/Supplementary Material, further inquiries can be directed to the corresponding authors.
