# Supplementary figures and images for: Tirzepatide ameliorates spatial learning and memory impairment through modulation of aberrant insulin resistance and inflammation response in diabetic rats
Source: Front Pharmacol. 2023 Aug 28;14:1146960. doi: 10.3389/fphar.2023.1146960 (PMC10493299; doi:10.3389/fphar.2023.1146960)

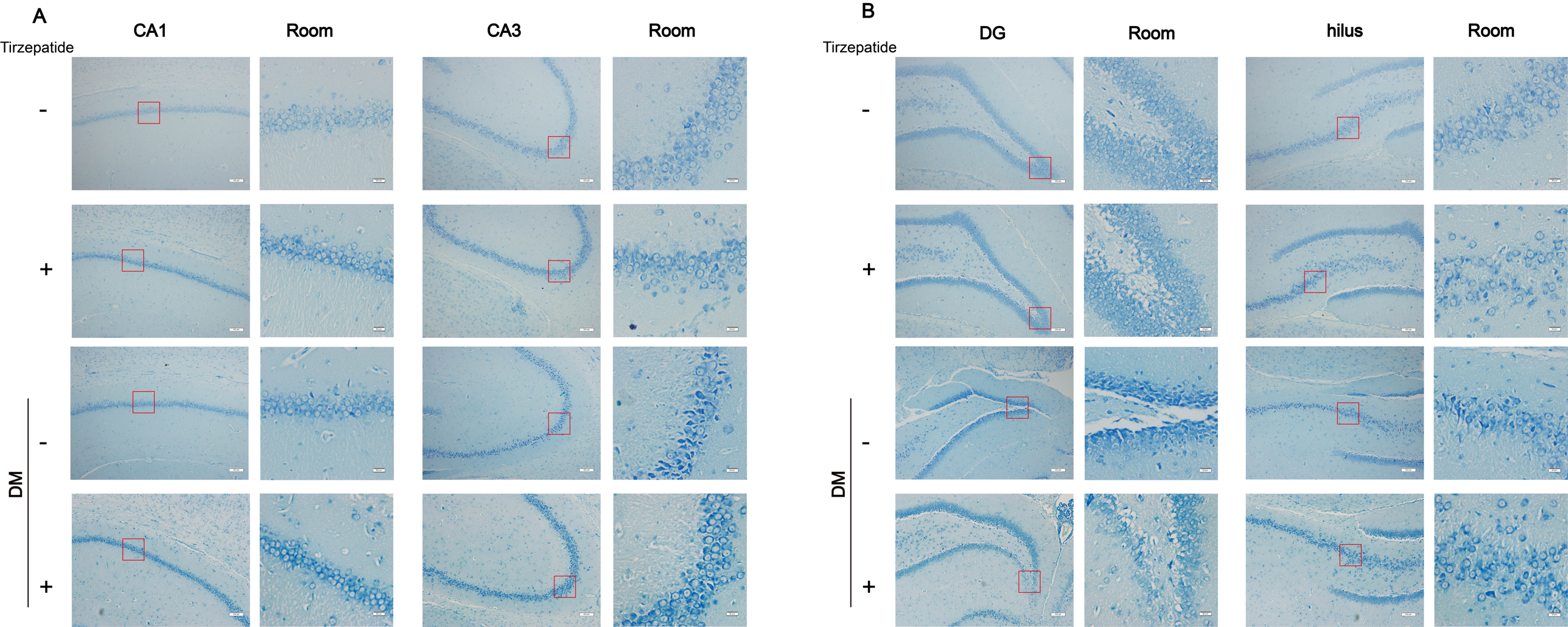

Supplement: Supplementary file 1 [file Image1.jpeg]
